# Identifying Leadership Characteristics from Social Media Data during Natural Hazards using Personality Traits

**DOI:** 10.1038/s41598-020-59086-0

**Published:** 2020-02-14

**Authors:** Amit Agarwal, Durga Toshniwal

**Affiliations:** Indian Institute of Technology Roorkee, Computer Science and Engineering, Roorkee, 247667 India

**Keywords:** Natural hazards, Engineering

## Abstract

With the availability of smart devices and affordable data plans, social media platforms have become the primary source of information dissemination across geographically dispersed users/locations. It has shown great potential across different application domains including event detection, opinion analysis, recommendation, and prediction. However, the process of extracting useful information from the collected voluminous social media data during natural hazards is a standing problem that needs significant attention from the research community. The fine-grained knowledge detailing users’ participation in information spreading could be advantageous in developing a reliable social network for the adverse events (Natural Hazards, Man-made attacks etc.). However, there has been no such findings related to identification of leader and their leadership characteristics associated with natural hazards in previous studies. We have collected 20.6 million tweets which were posted by 5.3 million users, during distinct devastating hazards namely - Floods, Hurricane, Earthquake and Typhoons. To achieve the goal, we divided our work in to three parts. Firstly, classify the collected crises data into four domains i.e resource, causality, news, and sympathy by employing deeper recurrent neural network model. Secondly, we used statistical physics of complex network to recognize local as well as global prominent leaders. At last, we curate leadership characteristics in terms of their big five personality traits and emotional traits. Our experimental, results find evidence that local leadership behaviour characteristics are significantly different from global potentials. Where as we also finds that some behaviour traits were certain to classified domains (resource, causality, news, and sympathy) and some were certain to hazard divisions, though emotional characteristics remained consistent. Later, we conclude that local potentials leaders have comparatively higher emotional strength. Furthermore, when the complete local network structure is unavailable, we find that the dynamic rank is reliable indexing proxy for local potentials. The current study, provide useful insight to understand how leadership characteristics are influenced to hazards, domains and centrality of users.

## Introduction

Nowadays, billions of active users over worldwide use social network platforms (SNPs) to interact with each other and shares their day to day activity over platforms like Twitter, Facebook, Tumbler and many more. SNPs have an ample amount of real-time information sources pertinent to real-world events, predominately during any natural hazards or mass crises. These platforms play a vital role to disseminate any information among users through online social interaction^1–3^. The previous study on information dissemination reveals that few influential knowledge spreaders (IKs) have the characteristics that can shape millions of users viewpoint^4^. Therefore identification of IKs holds immense practical importance and also in recent years it attracted the considerable attention^5,6^. So to identify the IKs, several distinct ranking algorithms were used previously. But these ranking algorithms are heavily dependent on numerous topological measures. For instance, the bidirectional link-based leader rank algorithm^7^ has a faster convergence rate than the widely studied page ranking techniques. However, inverse proportion involved in the leader rank technique makes him an inappropriate candidate who allocates higher probability flow score to a minimum leader node. Therefore, an efficient, effective and reliable influential predictor is needed in locating resourceful nodes for mass spreading. The most prominent and widely studied techniques include - In Degree, Betweenness Centrality, PageRank and K-Core Decomposition apart from the numerous identifying spreaders measure proposed over the years. There are a number of different applications were IKs can play a vital role as it gives acceleration to information diffusion in marketing applications^7^, identify top leaders during election^8^, leaders from criminal network^9^, identify the spreader during rumour and fake news^10^ and opinion leader in question-answer community^11^. Although IKs identified over the different applications, but there is an absence of identification of influential spreader and characteristic of these spreaders over natural hazard events such as - Hurricane, Earthquake, Typhoon, Flood. So in this work, we have identified prominent local leaders in various natural hazards (as our adverse events), i.e. refer to knowledge spreaders during the post-disaster events by using statistical physics of complex networks. Furthermore, we also categories leader characteristics by analyzing their behaviour as well as emotion characteristics. This kind of finding can be beneficial from SNPs, as it can provide the valuable insight during time-critical situations and also IKs helps to accelerate the information diffusion and awareness through their network (or links) to an affected community. It has been observed from the recent years that SNPs serving as a useful platform for time-critical situations or situational information during natural hazards^12–15^. Author^16^ have discussed the models and data of different events such as disasters, crime, war, and disease spreading to show how the traditional methods are not effective, sufficient as well as they have not providing the actual picture of the system. They have highlighted the a suitable system design and management that can help to stop the undesirable cascade effects. In^17^ have review the users social dilemmas that is authors have discussed the cases where the individual interests are different from the interests with others users, and also where AI might have a hard time while take a right decision as well as author also discuss the jurustic challenges.

However, previous work performed over crises have been divided into three categories, i.e. Firstly, to extract the tweets regarding natural hazards^12,14,15^ Secondly to match tweets relevant to information seekers and suppliers^18–21^ i.e. (Need and availability tweet) and at-last classify the tweets into different categories^13–15^ (casualty, resource, news, sympathy). In^12^ authors releases 7000 (EMTerms) emergency management terms that used in twitter over 35 different crises. The EMTerms classified into 23 information specific categories, with the help of experts annotators and crowdsourcing. Whereas in^14,15^ authors classify the tweets into personal, direct and indirect informative, and many others. In traditional approaches, the author used a set of regular expression(RE)^18^ and pattern matching^19^ to identify the tweets related to need and availability tweets, but these methods require a lot of manual work in preparing the RE and patterns. However, to overcome these issues, the authors proposed word embedding based retrieval method^20^ to improve the results as compared to traditional techniques. Few studies have shown, geospatial information diffusion over the globe based on re-tweet network^3^. After identification of IKs, we came to know that there is negligible systematic, data-based evidence has been published to guide leadership qualities in disaster-affected communities. So we further, find out the behaviour pattern by exploiting the personality traits of these leaders. Personality reflects the way users behave and reaction in SNPs and in society. Traits model extensively studied in psychology and social science. Numerous traits models have been proposed previously to determine personality traits, in which some of the most prominent and influential ones, i.e. Allport’s traits theory, Eysenck’s three dimensions of personality, the Myers–Briggs type indicator, and Cattell’s 16-factor personality but all these models have gained popularity from several decades. But with the recent advancements, big five traits models^22^ have emerged as widely acceptable and more reliable behavioural representative. It consists of openness, conscientiousness, extroversion, agreeableness, and neuroticism that were accepted as fundamental different and independent domain traits, that were emerged from previous personality test analysis^23^. Extensively research already had been performed to prove the validity of the model over different languages^22,23^. The extensive research has compelled a vast majority of psychology researchers to accept the big five model as of the current personality trait model. To the best of our knowledge, no one previously identified the behavioural and emotional personality characteristics of IKs from SNPs for various adverse events. A few research studies over SNPs has shown conscientiousness and extroversion traits were positively correlate with ease of social media use. Additionally, extroversion alone correlates with the size of the user’s social networks in various studies, and likewise, we have found out the leaders emotional characteristics over these social platforms. Previously, there was three most prominent and independent dimension of word meaning, i.e. valence (pleasure-displeasure), arousal (active-passive), and dominance (dominant-submissive). So in this study, we have chosen these independent dimensions and for that, we have used previously studied reliable 20,000 lexicons^24,25^. Although in recent studies, several authors manage to align these emotional characteristics to various genders. But none of the past studies has performed emotional traits analysis on these three dimensions (i.e valence, arousal, and dominance) to determine prominent characteristics of influential knowledge spreaders in adverse events. Likewise, previous studies in statistical physics of complex network, our outcome of extensive analysis reveal better effectiveness of the k-core algorithm for locating spreaders in adverse events as compared to page rank and in-degree centrality algorithms. The most influential knowledge spreaders are later filtered out with the proposed dynamic ranking expression. Our findings thus identify more efficient knowledge spreaders in determining their behaviour and emotional characteristics associated with adverse events.

## Results

### Construction of Natural Hazard Network

In this study, we derive the personality characteristics of most prominent knowledge spreader for each resource, causality, news and sympathy community groups. These inferences are estimated on combining past contextual information for behavioural and emotional traits of user groups in the respective devastating natural hazard category, namely, floods, hurricane, earthquake and typhoon. We have collected tweets of these hazards on mentioning trending hashtags by using Twitter Streaming API to retrieve collective response individually for a variable time frame based on popularity over SNPs. The restriction on individual tweet length, generally compels users to use abbreviations and shortened. So, to remove such noise, we process the entire hazard response using previously known methods^26–28^ (see sub-section “Inundation Modeling”) in our study. Additionally, imposing a strict threshold on maximum keyword per hazard (here, 4) aids in crawling most reliable tweets without losing much information. Since overlap of keyword with more than one event results in further uniform random sub-sampling^25^. Therefore, it is relevant to underline that mainly five continents are actively participating in following empirical study without compromising privacy concerns of contemporary users. We have only used English language tweets in this study. We summarize the characteristic distribution of all studied data in Table 1 while neglecting few missing tweets (for some days) due to minor connection errors. Details about these natural hazards are explained as follows.

- The terrible *Kerala Flood* taken place (on 9 August 2018), due to an excessive amount of rainfall during the monsoon season. It is so severe that over 483 people lost their life’s and 14 were still missing^29^. The government had estimated property damaged cost more than US $ 5.6 Billion. Several agencies have significant contributions to name a few - various forces of India, Red Cross Society, IsraAid, People’s Foundation and many more^30,31^. Most of the campaigns were initiated through SNPs and gained significant popularity over a period of 21 days from August 11th, 2018, comprising 443,591 users having 2,821,469 responses overall.
- The *Hurricane Florence* originated in the west coast of Africa on August 30th, 2018 and became category four hurricane (wind speed up to 130 mph). That cause severe damages, including economic loss of more than US $17.9 Billion. Likewise, a hurricane of category five takes more than 3057 human life that originated in the same coast on September 12th, 2017^32^ that reaches peak intensity on September 16th. It hits with a severe impact on Puerto Rico and Dominica location that causes more than the US $ 90 Billion economic loss in total^33^. Apart from countless support, US Armed Forces, Caribbean Catastrophe Risk Insurance Facility, Red Cross and many other agencies are majorly came forward to control adverse situations. On August 3rd 2017, a disastrous Hurricane Irma strikes at Cape Verde that reaches to a peak velocity on August 6th. After several variations in intensities category, five hurricane impact severely to the Caribbean and Continental US with $ 64 Billion economic loss (more than 154 fatalities). Additionally, we find that roughly 7,678,002 response was made on SNPs from numerous geographically distributed users.
- On November 12th, 2017 Sunni and Iraqi Kurdish city of Halabja felt at least 50 aftershocks due to the deadliest earthquake of 7.3 magnitude strike near to Iran-Iraq border that took almost 630 people life with $5 Billion EUR economic loss. To cope with the situation, neighbouring countries initiated the crisis management campaign along with international Red Cross society. Equivalently another 6.9 magnitude earthquake occurred at the island of Lombok that took more than 560 people lives on August 5th, 2018. We find that among others^34,35^ regional government majorly provided most volunteers and medical aids and approximately 4,493,488 response were made on SNPs to overcome the situation.
- With the 4,873,236 response on SNPs, the devastating *Typhoon Mangkhut* injured more than 200 people in mid-September of 2018. Even though the medical and emergency response team was on standby, still 260 people loss life and widespread damage cost crossed $3.74 Billion in the Philippines and adjoining regions of the South China Sea. Over 20 people lost their lives in similar category five typhoon (Jebi) in Japan on August 25th, 2018, but it underwent rapid intensification on August 29th that impact severely with $3.4 Billion economic loss^36^.

**Table 1:** The statistical properties of the novel and proposed corpus of Floods, Hurricane, Earthquake and Typhoon in this article.

Crisis	Location	Distribution		Date		Hashtags
		Tweets	Unique Users	Initial	Days	
Kerala Flood	Kerala, India	2,821,469	0,443,591	11-08-2018	21	KeralaFloods, KeralaRain, PrayForKerala, dukkidam
Hurricane Florence	Carolinas, USA	1,534,192	0,777,820	13-09-2018	8	HurricaneFlorence, Florence, Hurricane
Hurricane Maria	Porto Rico	3,656,968	1,377,291	16-09-2018	26	HurricaneMaria, PuertoRico, Maria Hurricane
Hurricane Irma	Continental US	3,867,842	1,511,843	07-09-2017	10	HurricaneIrma, Irma, IrmaHurricane2017, Irma2017
Iran–Iraq Earthquake	Iran–Iraq Border	3,414,299	0,917,071	13-11-2017	48	earthquake, quake, Iran, Iraq
Lombok Earthquake	Lombok, Indonesia	1,079,189	0,413,528	08-08-2018	17	Lombokearthquake, Indonesia, earthquake, quake
Typhoon Mangkhut	Hong Kong Philippines	0,429,215	0,255,681	15-09-2018	6	TyphoonMangkhut, Mangkhut, Typhoon OmpongPH
Typhoon Jebi	Japan	4,444,021	1,877,940	04-09-2018	17	TyphoonJebi, japan

In this table, location represents the reported place of hazard and the most prominent keywords were listed in hashtag column. The count of English language tweets (set of edges) along with a unique number of users (set of nodes) are reported. Besides, days indicates the interval of streams collected from the initial time-stamp. All corpus are available individually on request.

**Figure 1:**
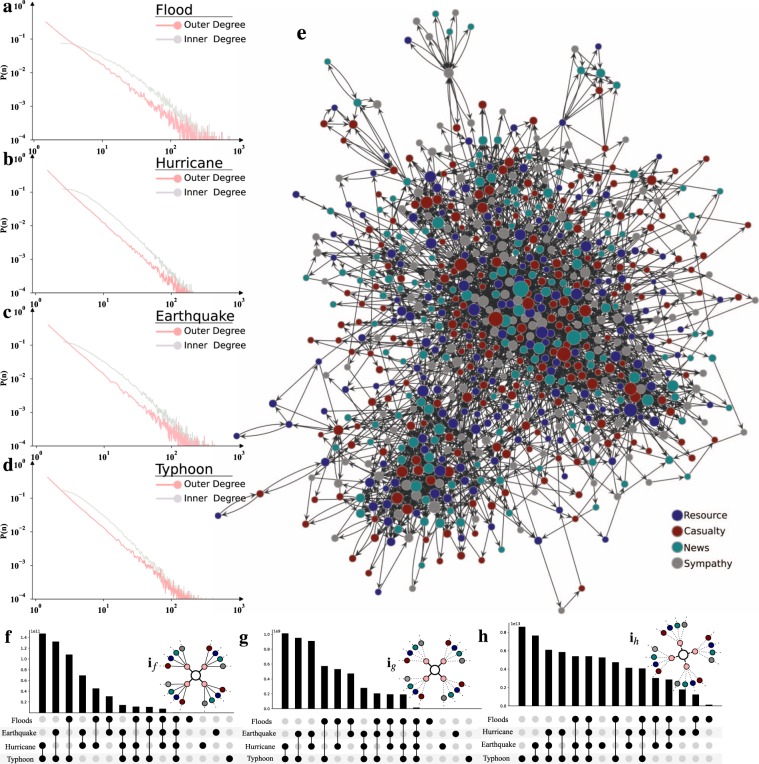
Construction of hazard response network based on type of interactions. (**a**–**d**) Distribution of degree strength for directed real network. A power law is necessarily a statistically plausible model of a network’s outer degree distribution with a strong constraint on alpha (i.e 2 $\ll$ 3)^66^, (**e**) The four distinct interaction types are represented in a component of the hazard response network and the color intensity of edges resembles the number of tweets involved in the direction of response the network graph created by using the Gephi tool^38^, (**f**,**g**) The presence of global users in different combinations of the networks. The group of nodes makes a presence in the interconnection network of hazard that includes all interaction divisions explained with smooth edges in (*i*_*f*_) and dash edges of maybe nodes as shown in (*i*_*g*_) represents the presence of more than one interaction instead of all, (**h**) The existence of candid users in distinct network groups, i.e combination of local leaders network illustrated in (*i*_*h*_).

As a result, we could use these responses to trace the type of information being conveyed by most influential users. We then build the hazard response network from users response following the reference methods formalized in^37^ (see Methods). In which single node represents a user of mentioned response such that a directed link between any two nodes is defined whenever response (here, tweet) of the node was re-responded (here, re-tweet) through another node. In the realm of a communication network, the resulted nodes with an outer degree (d) of a scale-free directed network follow a power law n^−a^ (see Fig. 1a–d) and therefore, these nodes are individually annotated with their major contribution among a various division of response. In the network of various hazards, for some response, we find many self-responded users who have a direct link with itself. But for all response, we find some users always prefer to re-response another node. Actually, they only promote the response of another user during the period of time we collect data. Assuming such response are absurd for hazard response network, we exclude the response as well as a node (in absence of a response) from the network. In all networks of devastating hazard, we only consider the largest connected component. A hazard response network is drawn by using the Gephi tool^38^ as shown in Fig. 1e. In addition to it, we organize three different definitions for these nodes (see Methods) illustrated in Fig. 1 *i*_*f*_, *i*_*g*_, *i*_*h*_ to find their presence in different combinations of hazard network. Across these combinations, we find robust evidence of activeness level and major curiosity of these nodes representing a unique user as described in 1f,g,h and for some groups combinations have significant overlap in response,representing global potential leaders as shown in Fig. 1f,g. However, we are specifically focusing on local potential leaders (Fig. 1h) to provide further essential findings.

### Region of Interests

**Figure 2:**
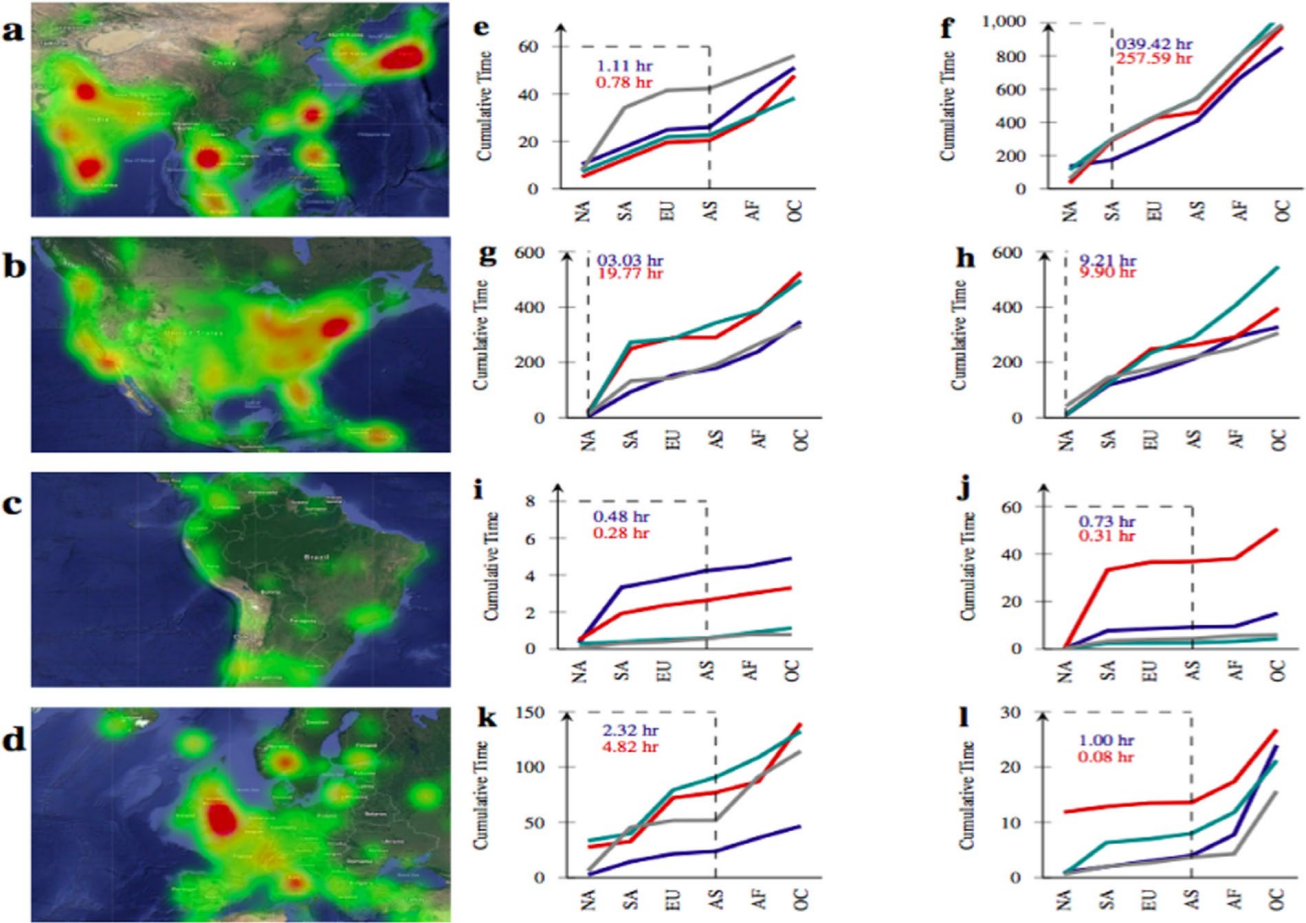
Schematic illustrations for the region of interest and feasibility in response hazard networks. (**a**–**d**) Collective geospatial response intensities over most active regions and we have used the orange tool^67^ to plot coordinates over the map. The frequent sub-region intensity is highlighted with red colour and lesser with green colour, (**e**–**l**) The various introductory response beginning from the formation time frame of a hazard and arranged as per sequence in Table 1. Here, 11.36 is the network feasibility of resource response and 5.97 for casualty domain. Lower feasibility score has greater support by local leaders in all cases. The y-axis represents the cumulative time in hour scale and dashed line represents the earliest responded region (mentioned with values as well), (**e**–**l**) NA represents North America, SA represents South America, EU represents Europe, AS represents Asia, AF represents Africa and OC resembles Oceania region respectively.

The diverse population of geographical regions and sub regions reveals significant contributions in the time frame of natural disasters^39,40^. Among them, Asia, Europe, North America, South America and Australia made most need contributions, specific sub-regions are highlighted in the Fig. 2a–d. The relief contributions from the neighbouring sub regions in flood network have higher distribution comparative to various tier-I sub regions and as an instance, a high mobility indexed sub-region (tier-II^41^) express higher response and users from a best-listed region^42^, Kansas county contributed maximally to all networks of hurricane. Additionally, we find that vertices of the network are more frequent towards casualty response than news. Likewise for hurricane located at Carolinas and typhoon mangkhut as well but it advances to news response on increment to damages and fatalities. The distribution of Lombok earthquake network vertices is statistically higher from central Kalimantan (including neighbouring sub-regions) for resource interactions and partially remain same on combining the remaining earthquake network. Lastly, we conclude that users from kansas county region have significant reachability in real social networks which enables them to contribute in various hazard networks.

### Response classification for network feasibility

The need for determining feasibility in response hazard network is due to the inclusion of overlapping sub-events irrespective to study domain^43^. The removal of response created before the formation timestamp of a natural hazard (available on wikipedia publicly) can avoid this false consideration to some extent. These segregated responses are classified and later, cross-validated with the various news articles (published at well-known venues) to strengthen the fact that early forecasting of natural hazards triggers various disaster response forces, to combat life-saving situations. We empirically find that most of the time the resource response commences first in the network instead of casualty and surprisingly within 24 hours from the formation hours as illustrated in Fig. 2e–h,k,l, specifically for long-range predictable natural hazards (Floods, Hurricane and Typhoon^44–47^. The classified interactions in a network also reinforce that the location of a preliminary resource response is identical to the continent of predictable hazard. Furthermore, the neighbouring regions (even sub-regions) also support widely in minimizing the fatalities during a short span predictable natural hazard (see, Fig. 2i,j) i.e earthquake. For the users involved in response hazard network ($\Gamma$), we calculate the network feasibility M($\Gamma ,\varphi ,\phi$) with a given severity timestamp (*θ*):

1 $$M(\Gamma ,\varphi ,\phi )=[\sum _{i\in \Gamma ,j\in \phi }\,({\varphi }_{i,j}-{\phi }_{i})]\times \frac{1}{{\Gamma }_{n}}$$

Here $\phi$ represents the introductory response timestamp of interaction $\phi$ in the network. Then we transform observation in hours scale and display them in Fig. 2e–l. It is observed that for the introductory response of casualty, the feasibility constant is relatively higher for the long span estimated hazard while lower in short span predictable hazard. Furthermore, we find the initial response of news is positively correlated with the resource, only in case of long span predictable hazard and the hazard response network was flooded with mixed interactions (except resource) in short span predictable crisis. Comprehensively these empirical observations resemble the reliable feasibility of the networks.

### Keywords classification for model feasibility

To eliminate the dependency of cross-validating the domain labels of these streams, we archive all the relevant news articles published from the formation date of a hazard for a period of one month in a single document. The studied articles are publically accessible, can be again archive with the hashtags mention in Table 1. We normalize all these mentions (available in the document) to a lower case and later, remove the occurrence of described annotated lexicons (see methods) that include commonly used stopwords of English literature as well. Then we estimate the number of uni-grams which actively participated in the categorized response of hazard as well as in processed articles. The distribution frequency of these influential uni-grams represents the overall feasibility of the model. The precision of the model is feasible only when feasibility ratio (f) between uni-grams of articles (*α* set) and the intersection of them with specified class of response (*β* set) are closer to 100 (and error closer to 0). We define the feasibility ratio f(w) as

2 $$f(w)=\frac{|\alpha (w)\cap \beta (w)|}{|\alpha (w)|}\times 100$$

We find the archived uni-grams are more biased towards news and sympathy interactions, possibly due to a specific focus area and presence of short text languages. Furthermore, the subform of volunteers, clothes, food packets and small variations in aid labels were the top-ranked uni grams in the model while fatalities, found, rescue and life were the most influential uni-grams of resource and remaining interactions respectively

### Algorithmic influential users measure

**Figure 3:**
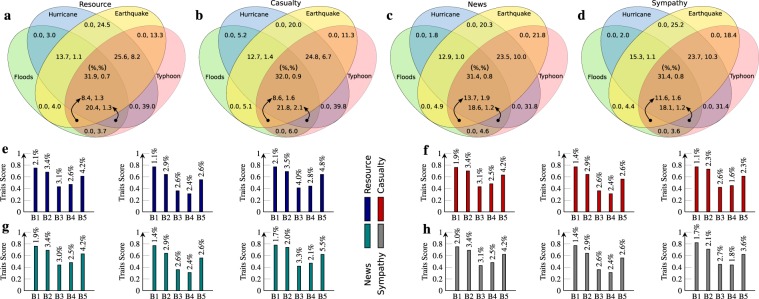
Skewed overlapping distribution with the behaviour of global potential nodes. (**a**–**d**) The division of nodes are segregated based on the type of interactions in a hazard response network. The percentage of significant overlap in nodes is shown inside eclipse in which maybe nodes represents initial while remaining represents unique potential nodes distribution, (**e**–**h**) The sub-panel 1 and 3 illustrate the personality traits on the y-axis of above nodes, whereas 2 represent for the common potential nodes. Here, B1-5 symbolize Openness, Conscientiousness, Extraversion, Agreeableness, Neuroticism traits respectively on x-axis. Numerical value on bar top means standard deviation. The behaviour patterns (B3-4) in extreme left and right sub-panel are analogous to each other except sympathy response but get reverse with common potential nodes.

The characterization of users in the network (see, graph 1e) reflects transparent usage of SNPs in various natural hazards and exploiting such dynamics are capable to eliminate the model-specific dependency on finding influential users to some extent. But it demands the dynamics of entire network structure, though absent due to the presence of various privacy policies of users. Consequently, their absence creates situation worst on further segregating influential users majorly based on interaction type to its peers. To better understand the importance of local influential user, we illustrate the dynamics of global nodes in Fig. 3a–d. (described with initial percentage value). The presence of significant overlap among hazard divisions determines that the potential uniques nodes are more influence on resource and casualty than news and sympathy but common nodes, are less influenced to resource interactions. In addition to the response (see Fig. 1f), the potential common nodes are more frequent to hurricanes and typhoon as well. Therefore, we determine the candid nodes are more favourable to be influential due to (almost) equal response distributions. We also provide better insight through big five personality model (see methods) traits of these global nodes as described in Fig. 3e–h, to form a baseline for local influential users. We find the presence of a higher significance level for openness traits over these global potential nodes, though remain maximum with unique potential. The higher significance of conscientiousness traits indicates nodes interaction are more favourable to share precise information. These nodes are analogous to extraversion traits which signify the presence of acquaintances in the actual network although it remains insignificant to common potential nodes. As stated in the literature, agreeableness trait is favourable of happiness of others but global potential nodes are lesser relevant to the same behaviour although relatively higher neuroticism trait, enables more stability and emotionally resilient. Therefore, it becomes essential to strongly validate the outcomes that are highly sensitive to the mathematical assumptions of the underlying model.

3 $$SF({k}_{frac})={\log }_{10}[\sum _{i\in {k}_{frac}}\,\frac{S{F}_{i}}{N({k}_{frac})}].$$

**Figure 4:**
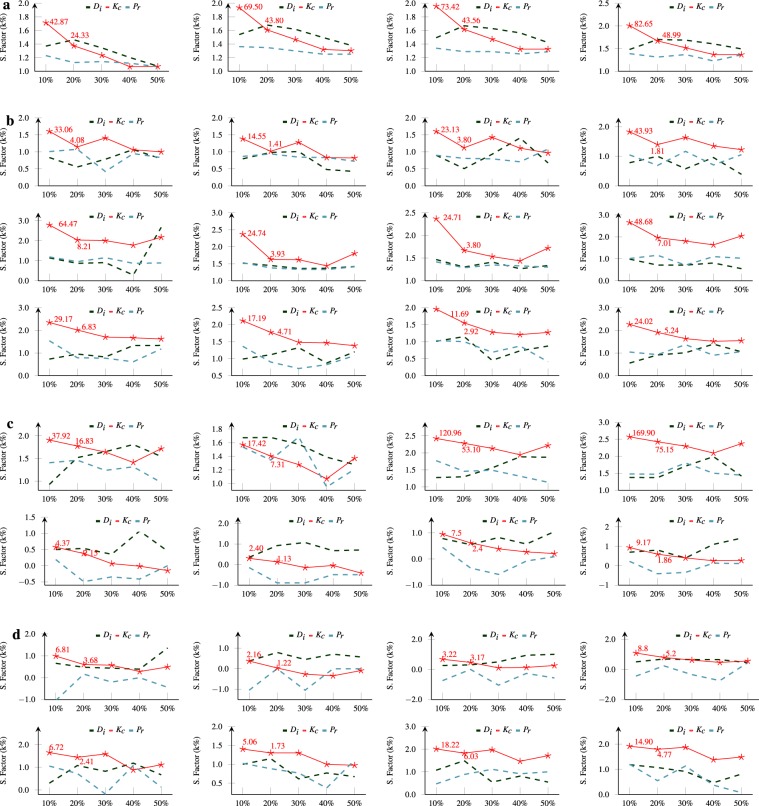
The nodes with lower inner and proportionally higher outer degree have a higher spreading factor. The most prominent nodes following graph decomposition method is a group within the initial fractions of users, (**a**–**d**) Here, 116.40 is the overall spreading factor for bins of most promising users. The nodes are arranged as per sequence in Table 1 where leftmost subfigure describe observations for Resource, Casualty, News and rightmost represents Sympathy. The y-axis describes the logarithm value of spreading factor and x-axis represents fractions of users aligned with the dynamic rank measure. The values on top of initial bins describe standard deviations of the underlying fraction.

**Figure 5:**
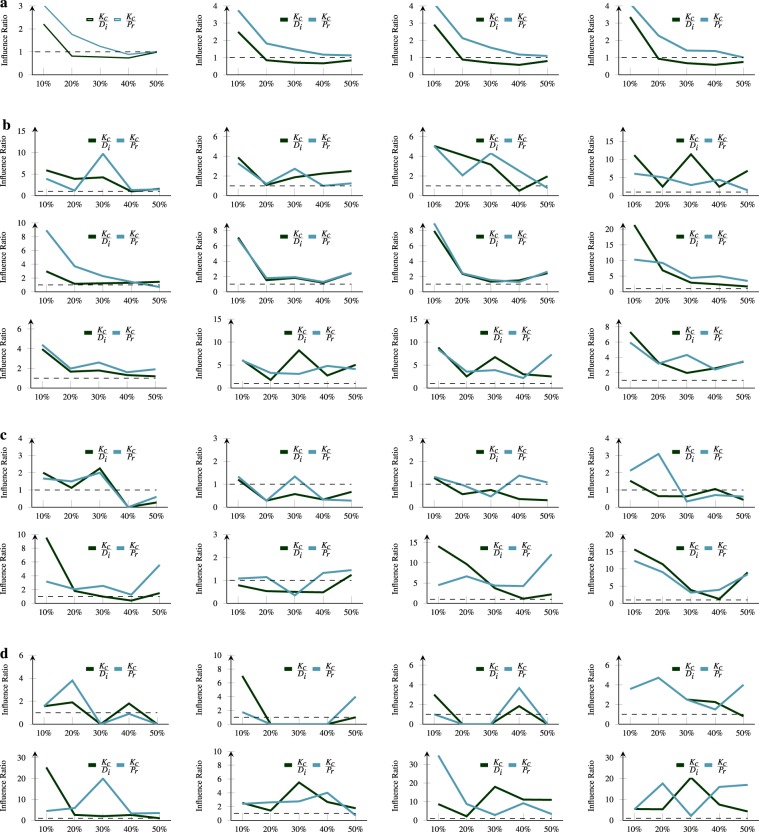
The k-core (*K*_*c*_) has 6.42 times higher influence ratio than degree centrality (*D*_*i*_) while 5.47 times higher than page rank (*P*_*r*_) measure in initial bins. The baseline observation is described with a dashed line that marks the positive ratio 1. (**a**–**d**) The average influential ratio represents the proportion between mean spreading factor of *K*_*c*_ within fractions (describe on the x-axis) and that of the other two measures. Likewise, panels are arranged as per sequence in Table 1, except for observations in the earthquake panel where leftmost subfigure describe observations for Resource, Casualty, News and rightmost represents Sympathy.

We calculate the average spreading factor SF (${k}_{frac}$), purposely for a top fraction (here, 50%) of local potential nodes in hazard response network and then arranged with given indexing measures (see Methods). Here ${k}_{frac}$ is the collection of all the nodes participate in spreading and N(${K}_{frac}$) represents the total count of these local potentials. Later we take their logarithmic value (base 10) and display them in Fig. 4. It is observed that for nodes with a lower inner and proportionally higher outer degree, SF will be higher (excluding boundary conditions). Additionally, we find the nodes located in the lower shell tend to have a lower rank. We compare the variation in spreading factor for nodes within a fixed set interval on the x-axis to have a direct view of these observations and found k-core decomposition (*K*_*c*_) remains on top. We divide the range of measures into five distinct bins equally to the distributions of non-leaf nodes in hazard response network and then calculate the factor SF, for nodes within each bin. Consider the page ranking algorithm (*P*_*r*_, see Methods) as an example, we create five separate fractions (i.e bins) of ordered nodes, arranged as per page ranking score. In Fig. 4a–d, we find that the standard deviation of spreading factor is higher for an initial fraction but comparatively lower for remaining bins. Although deviation gradually decreases with a reduction in the factor of bins. This means that spreading factor of nodes in initial bins were not random i.e. most influential nodes are a group within initial bins in hazard response network. Therefore, Fig. 4 shows comparatively higher performance with graph decomposition method (*K*_*c*_) in comparison with widely studied page ranking and degree centrality algorithm. The spreading factor of *K*_*c*_ is dominant from the initial bins onwards. However, in some events (see Fig. 4c,d), the degree centrality (*D*_*i*_) become superior with a small factor with the due absence of required participation We find that the overall spreading factor in a hazard response network is 116.40 for bins of most promising users, comparatively less than a network^37^ evaluated on the inner degree. It means in a real scenario, the outer degree of nodes in hazard response network always be lesser than the inner degree and largest connected component of a network will never be able to cover more than half of nodes irrespective to a number of fatalities and damages. Additionally, the higher distribution of smaller disconnected components from the giant leads to the formation of fewer shells. We find that compared to various interaction in the network, news and sympathy have higher influence. The gradual increment of user bins in *K*_*c*_ results in a decrement of an overall spreading factor on the progressive selection of smaller factors. This formation is reliable, but a similar formation of spreading factor is absent in page rank and degree centrality. To further formalize the reliability of initial *K*_*c*_ bins, we illustrate the average spreading factor ratio of it among other measures in Fig. 5a–d. The ratio consistently remains to be larger for initial nodes bin’s, compared to both measures. This means the initial bins with high spreading factor have the most influential nodes arranged via *K*_*c*_ measure because the reverse of the ratio has negative significance with page rank and degree centrality measures. The influence ratio of *K*_*c*_ is ten times higher than of degree centrality (*D*_*i*_) and more than eleven times larger than classical page ranking measure, as shown in Fig. 5d. Additionally, we also find that on an average k-core spot six times higher interactions than degree centrality in initial bins. Likewise, it comprises three times higher factor in comparing the distribution of users with page rank measure. Though a significant reduction in these factors on transitioning to higher bins but still graph degeneracy methods attain higher factor on comparing with degree centrality as well as page rank algorithm indicating presence of threshold in finding most influential users. Lastly, we find that overall *K*_*c*_ has 6.42 times higher influence ratio than degree centrality while 5.47 times higher than page rank measure. It implies in most cases the nodes with higher spreading factor always accumulate in the initial bin’s only because the transition towards adjacent bin there is a reduction in stated value by 61.05% and 34.55% respectively.

**Figure 6:**
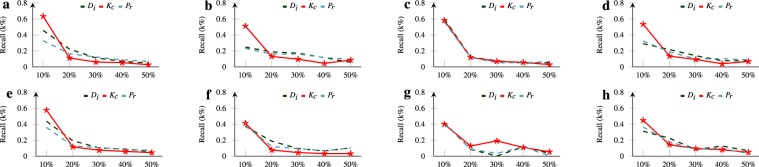
The recall for *k*_*c*_ is larger than page rank and degree centrality. Robustness in natural hazards events <Resource, Casualty, News and Sympathy>, (**a**) Kerala Floods, (**b**) Hurricane Florence (**c**) Hurricane Maria (**d**) Hurricane Irma, (**e**) Iraq Earthquake (**f**) Indonesia Earthquake (**g**) Typhoon Phili (**h**) Typhoon Japan.

Though *K*_*c*_ can estimate the mean spreading factor of nodes well in initial bin’s, whether it can better locate individual (or group) influential nodes are still not clear. Therefore, we evaluate the recall for these bin’s and determine the performance of measures with the most influential nodes. We define recall as:

4 $${Recall}=[\sum _{i\in \alpha }\,\frac{|{N}_{i}\cap {k}_{frac,i}|}{|{N}_{i}|}]\times \frac{1}{\alpha }$$

where *α* represents the cluster of collective nodes of various interactions though arranged with stated measures only. The ${k}_{frac}$ represents the set of nodes available in the initial bin of sub-network and N expressed as the cardinality of the set. In Fig. 6, we illustrate the individual bin’s recall of various hazard response network and finds the recall for kc is larger than stated measures. Though degree centrality and page ranking measures have a similar recall as *K*_*c*_ still these are more vulnerable to randomness due to high fluctuations in the remaining network (see Fig. 6a,b,d,e,h). Additionally, we find a hazard response network of Kerala has a uniform recall across various sub interaction network. We also find the Kc has a maximum recall (average 51.17%, see Fig. 6) while focusing on initial bin’s of the various network. Likewise, we find the similarity in recall among categories of various hazard. Therefore, the proposed measure is capable to find more reliable influential nodes than degree centrality and page rank and with robustness in predicting information spreaders, it enables the nodes of a network to attain higher influence ratio. Further literature on the invalidity of degree centrality and page ranking measures can be found in^48,49^. Thus, the initial fraction with 10% threshold is among the best in estimating the topmost knowledge spreaders.

### Behavioral and emotional personality traits

We have already seen that for most obvious candidate, the node out-degree alone is enough for identifying the initial fraction of local information spreaders. It’s reasonable to assume that the more efficient spreaders are the ones who have not only high out-degree but their personalities also resemble high leadership traits. Indeed, this reasoning can be further generalized with behavioural and emotional characteristics respectively. These traits provide a too coarse description of influential users personality in terms of widely studied big five attributes. In addition to these adequate personalities, we more specifically measure the traits on various dimensions of these facets to describes the higher sub-relevance because various research on leadership emergence^50^ identifies numerous factors associated with the candidate, being perceived as influential. We find that the nodes from initial fractions have high behavioural traits and these nodes also have a higher degree of emotional characteristics in terms of positiveness, activeness and dominance.

**Figure 7:**
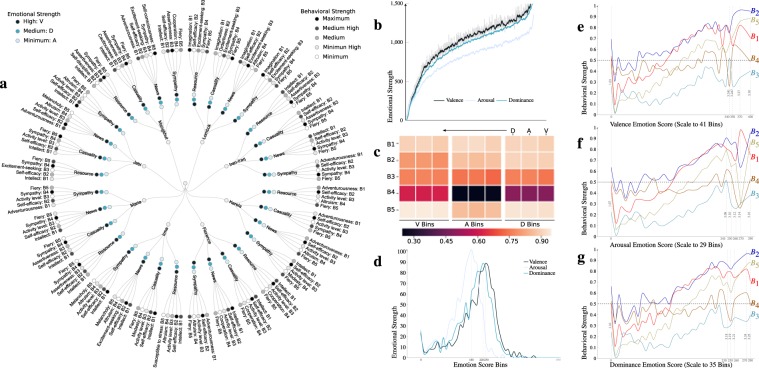
The prominent candid leaders from casualty and resource domain are more influenced to agreeableness characteristics. These behavioural characteristics of leaders are more analogous to maybe and unique potentials users, (**a**) Circular representation describes emotional and behavioural strength for prominent leaders where higher intensities represent maximum while lower with the minimum value, (**b**) The y-axis represents emotional strength of collective prominent users as represented on the x-axis, (**c**) The colour intensities represents Pearson correlation among emotional and behavioural characteristics. The emotional scores are segregated into dominance (D), arousal (A) and valence (V) bin’s where each sub-panel follows the same sequence, (**d**) Collectively, the distribution of individual emotional strength over bin’s are shown on the y-axis, (**e**–**g**) The behavioural strength are described on y-axis for individual bin’s and the horizontal dashed line represents threshold of average tendency for a characteristics where extraversion trait (B3) always lies in lower region.

These users have a higher shared understanding of intellectual curiosity for news interactions while imagination (a facet of openness attribute) has a factor to resource, casualty and sympathy individually. The cautiousness (a facet representing conscientiousness trait) attribute has a higher factor in casualty interaction among various other acknowledgements. The most appropriate candidate for resource response has a higher factor in an assertiveness attribute of extraversion characteristics. Simultaneously for an excitement-seeking facet, we find that casualty and sympathy response of similar fraction of users have a higher factor while agreeableness facet has a higher constituent in terms of the empathetic characteristic in sympathy interactions. Likewise, at the classification level of various hazards, we find that the most influential users from the floods are more likely to be self-assured and deliberate in a different set of users representing typhoon hazard. Interestingly in case of an earthquake, we find that the influential users are more likely to be imaginative, philosophical, excitement-seeking as well as empathetic. Though we find higher assertiveness characteristics in typhoon and hurricane users respectively but still cautiousness and sympathy characteristics remain higher. The remaining dimensions of these big five characteristics either have a low behavioural score or least frequent to most influential users fraction. However, the remaining dimensional intensity for the response in various hazards is summarized in Fig. 7a. After aggregating the strength of big five personality attributes, we find that openness and conscientiousness traits remain common (as well as higher) to all classification of response. The agreeableness characteristics have higher trait score among users from the resource as well as casualty and similarly in news, we find neuroticism characteristics to be higher. However, we find that sympathy remains ambiguous in terms of personality behaviour. Furthermore, we also find these users have a higher shared understanding of dominance and valence attribute on comparing with arousal emotional personality trait as described in Fig. 7b. Additionally, for a single response, we also find emotional traits were higher in sub-network of floods as well as an earthquake.

To evaluate the relationship between these emotional and behavioural personality traits, we formed three distinct hash tables with similar hash key (i.e 10) representing individual emotional traits. Later, we calculated the average behavioural score to represent each bin’s strength (outline on the x-axis and y-axis, see Fig. 7e–g). We analyse that the valence emotional score of the most influential users has maximum bin’s (41, representing high variance) but lesser with arousal score (29, representing least variance). Likewise, we analyzed the valence bin’s range from 0 to 410 but it gets reduced to 26% in emotional score with arousal (see Fig. 7d). We find that the behavioural personality score increases with an increment in bins for valence, arousal and dominance traits respectively (see Fig. 7e–g) and the emotional personality traits for some classification of response impact the strength of behavioural personality traits. For example, consider resource response, neuroticism trait strength is comparatively lower. For the most obvious candidates, we find the high correlation of valence and dominance trait with neuroticism characteristics (Pearson correlation, r = 0.92+) as illustrated in Fig. 7c. Additionally, these emotional traits also have a significant correlation with openness, conscientiousness, extraversion (0.72+ to 0.90) respectively. Similarly, openness has a significant relationship with arousal trait score, as compared to agreeableness score with valence, arousal and dominance characteristics.

## Discussion and Conclusions

Understanding leadership characteristics from distinct natural hazards are critical for the emergent warning, response and recovery management during a disaster. In this study, we have built a systematic framework for the trait analysis of local and global potential users in resource, casualty, news and sympathy response domains towards natural hazards. By evaluating the prominent geographical regions and feasibility of interactions among users in individual hazard response networks, we have shown distinctive sub-regions that are most frequent to Floods, Hurricane, Earthquake and Typhoons. Additionally, these users reflect the significant presence in the network and neighbouring sub-regions provides a higher relief contribution in most cases. By applying leaders identification algorithms from statistical physics, we find that k-core decomposition is more robust and reliable among the examined ranking measures. Further, the behaviour of potential indexing users in initial bins is far from random due to high spreading factor and robustness. In close relation to the evaluation of potential users, the content of response interact within these networks has shown significant influence ratio among news and sympathy. The k-core in hazard response network spot six times higher interactions than degree centrality in the initial bin’s and three times are higher in comparing the distribution of users with page rank measure. Finally, by comparing the robustness, i.e. the higher spreading factor interval, we find that the initial fraction with 10% threshold is among the best in estimating the topmost knowledge spreaders.

Moreover, our finding also demonstrates that the prominent local users have a higher shared understanding of intellectual curiosity for news response while imagination has a factor to resource, casualty and sympathy individually and it also indicates that excitement-seeking most frequent to casualty. Additionally, the potential local users from the typhoon are deliberate, assertiveness and cautious while assertiveness, cautiousness and sympathy for a hurricane, though imaginative, philosophical, empathetic frequent to the earthquake. Our results validate that the personality characteristics of prominent candid users are analogous to maybe and unique potentials users. Although these traits are not similar among common potential users, specifically extraversion and agreeableness behaviour strengths are different. It is worth noting that even though we used various hazards streams to increase the validity and reliability of the study, neither the training nor testing corpus is focusing on gender-specific characteristics. Therefore, future researchers are encouraged to use a gender-specific distribution (and various remaining profile attributes) to further increase the validity of their analysis. At last, we also conclude that in practice the dynamic ranking measure can be used to search for prominent candid users in the network since resultant users have higher valence and dominance score.

## Methods

### Inundation modeling

With a classification resolution up to four categories, recent event-based deeper recurrent neural network (DiRNN)^51^ model has received much attention for long sequence classification and have been established as an important source in large-scale information spread assessment. Following this approach, we make use of DiRNN to classify event streams of widely used microblog network for various devastating natural hazards. In addition to the initial parameter setting, a number of 256 hidden units and similar batch size were used as a modified hyperparameter of the model to support streams comprising numerous short text languages. To exploit the presence of independent neurons in each layer, the stacked network of model has been trained with the human annotated response of past equally-likely events in identical microblog network (see Supplementary Table 2). These labelled streams were mainly categorized into four distinct divisions for covering numerous aspects of available response, namely - resource, casualty, news and sympathy. The above-mentioned responses, as well as captions of images wherever available, were annotated by a group of independent paid volunteers to minimize human errors. Therefore a consequence of these gold standard annotations, the response having Sympathy only when previously classified labels contains following keywords namely - sympathy and emotional support, caution and advice, personal updates, praying and additionally some neutral keywords as well such as - not related, irrelevant, non-government, non-informative and can’t judge. The response of event streams was classified to Casualty only whenever assigned annotations were affected individuals peoples, casualties and damage, deaths reports, displaced people and evacuations, infrastructure damage, utilities, injured and dead people, missing trapped, found people, vehicle damage. Animal management, disease signs symptoms, disease transmission, infrastructure, not physical landslide, people missing found or seen, physical landslide, response efforts, traditional media, missing, trapped or found people keywords were categorized into news response category. Likewise, events of streams were categorized with Resource division whenever annotations consist of keywords similar to availability, volunteer, professional services, urgent needs, treatment, shelter and supplies, rescue volunteering, donation effort, requests for help, needs, money, humanitarian aid provided, donations of supplies, volunteer work, money, goods, services and some neutral keywords - informative: indirect, direct, indirect and source. Therefore, we build a training set of tweets with four divisions: 1) Resource, 2) Casualty, 3) News and, 4) Sympathy. We have used an uneven proportion of various tweets for each response class, totalling 8031 tweets for the resource response class, 7885 tweets for the casualty response class, 5774 tweets for the news response and, 12490 tweets for the remaining response class. The unstructured tweet content is tokenized to extract a list of reliable words on removing non-ASCII characters and stopwords. Although only english language tweets were collected still to avoid any worst we transliterated them into english language and replace all question mark, links, hashtags, mentions and numbers by the separate individual tokens^21^. Therefore, employing static DiRNN modelling on streams (see Supplementary Table 2) we estimate the classification category of various devastating natural hazards streams of floods, hurricane, earthquake and typhoon. The performance of various supervised classification models^52–54^ is tested with different hyper parameter settings and optimized is performed on varying batch size of each model individually. We find that the best score outperforms all the state-of-the-art classification while maintaining 74.875% average accuracies. Therefore, this inundation modelling effectively provides estimates in the categorization of actual community response available in natural hazard streams.

### Identification of cities from tweets

We use the systematic procedure^55^ to identify the spatial extent of continuous hazard streams to ensure consistent identification of cities. We thereby follow the definition of individual spatial units^56^ to identify (and allocate) nearest geographical polygon from the spatial information. These frequent polygons are highlighted individually in Fig. 2. Though, it disregards the effect of spatial information absence in a tweet. The coarse refinement of self-reported location in response may often project to the Open Sea, Antartica and Island regions and to avoid, we are focusing on seven continents using additional polygon vectors of cities^57–60^. It retains those refined locations that lie behind or overlap with the geographical polygon region. As a consequence, we restrict to majorly contributed cities and select 1043 cities for further investigation. The most frequent geographical region per continent are elaborated in Supplementary Table 1.

### Identification of Influential Users

The influential nodes of hazard response network have lager access to the authentic information about an event which provides asymmetric influence on others. These nodes reflect innovative individuals whose information broadly relies on vast interconnections. Often, these are not the first individual who initiates the management but likely to remain in topmost position. The most similar behaviour is described in the following literature^29^. These influential nodes are majorly classified with three broad divisions namely, (1) Candid: The locally influenced node are active to a specific hazard response network (further elaborated with a dashed line in Fig. 1*i*_*h*_), (2) Maybe: The behaviour of nodes enables them to make a presence in all networks, though it restricts to some interactions only (further elaborated with smooth and dashed line Fig. 1*i*_*g*_), (3) Potential: The nodes contributions are significant in to network hierarchy (formalize in Fig. 1*i*_*f*_). It can be further segregated based on interactions, classified as follows:

- **Unique**:The major interactions are restricted to a specified division.
- **Common:** The global node activeness is significant to all interactions. The group widely comprise numerously journalist, public figures and non-governmental organizations and many others.

The stated classifications have been studied earlier in educational social networks (refer^61^) though it has the potential to portray leadership behaviour in hazard response network as well. The typical workflow of k-opinion leaders identification algorithm initiates with the formation of various sub-clusters in hazard response network and later, terminates with the clusters arranged in higher sequential order based on prescribed relevance score. As a consequence, the topmost users will be declared as influential nodes. To explain their efficiency, we compare the performance with widely preferred page ranking and degree centrality algorithms on hazard response network. The algorithm provides a high ranking score to weakly connected neighbours and established with a presence of random information diffusion in the network. The ranking relation heavily dependent on outer degree, inner degree neighbours, epsilon and damping factors. More elaboration on these algorithms was discussed in the following literature^3,5,37,48^.

### Indexing of Influential Users

The dynamic ranking method follows a greedy approach in evaluating the maximum contribution to various classification categories relative to the count of re-tweets. This dynamic factors can be further generalized to include a favourite count, likes of the tweet and so on.

5 $$DynamicRank(user,u)=\,{\max }\{\frac{{R}_{u}}{{e}_{(R,u)}},\frac{{C}_{u}}{{e}_{(C,u)}},\frac{{N}_{u}}{{e}_{(N,u)}},\frac{{S}_{u}}{{e}_{(S,u)}}\}.$$

In a processed sample of the network, we estimate the dynamic rank of a user(u) based on maximum relative frequency of response with the corresponding dynamic factor(e) for the same set of tweets because a response is relevant only if the response is influential to at least single user apart from itself. Here, the various classification categories of a user’s response were represented with *R*_*u*_, *C*_*u*_, *N*_*u*_ and *S*_*u*_ constants respectively and the dynamic factor denotes the count of a given attribute for the same set of user response. However, due to resource constraint, we consider the in-degree of a node (signifies a user ‘u’) as a dynamic factor in a sample network.

### Personality modelling

We have used pre-trained behavioural and emotional personality models^24,62^ to determine the well-studied characteristics of influential users. The open vocabulary-based approach, tokenize the recent user response (here, maximum 3,000) to form n-dimensional vector representation of tokens^63^. Later, the author’s estimates attribute score through classical non-linear gaussian processes model with the trait scores of numerous twitter feeds generated from their conducted surveys. This demographic independent model archives 25% smaller absolute error from the state-of-the-art approaches and determine any behavioural score at or above 0.75 indicates observable aspects of the personality. Likewise, the demographic dependent emotion-based personality modelling represents the commonly used lexicons in twitter feeds into three independent dimensions, namely valence, arousal and dominance. The score of these dimensions was normalized using the best-worst scaling technique to addresses the curse of conventional rating scale methods^64,65^ namely, inequalities with same and different annotators, self-preference to a portion of scale so forth. Lastly, these stated personality scores were normalized to a scale of 0 and 1 using classical min-max normalization function whenever a group of users needs to be study collectively.

### System setup

This work is Implemented on Pytorch, Python3.5, Intel(R) AI DevCloud, 48 cores Intel Xeon processors, 96GB RAM on Fujitsu R930 workstation.

## Supplementary information

Supplementary Information.

## References

[1] Qi, J., Liang, X., Wang, Y. & Cheng, H. Discrete time information diffusion in online social networks: micro and macro perspectives. *Sci*. *Reports* **8**, 10.1038/s41598-018-29733-8 (2018). 10.1038/s41598-018-29733-8 PMC6082892 30089814

[2] Yoo E, Rand W, Eftekhar M, Rabinovich E (2016). Evaluating information diffusion speed and its determinants in social media networks during humanitarian crises. J. Oper. Manag..

[3] Cvetojevic S, Hochmair HH (2018). Analyzing the spread of tweets in response to paris attacks. Comput. Environ. Urban Syst..

[4] Katz, E., Lazarsfeld, P. F. & Roper, E. *Personal influence: The part played by people in the flow of mass communications* (Routledge, 2017).

[5] Ahajjam, S. & Badir, H. Identification of influential spreaders in complex networks using HybridRank algorithm. *Sci*. *Reports* **8**, 10.1038/s41598-018-30310-2 (2018). 10.1038/s41598-018-30310-2 PMC6085314 30093716

[6] Taha K, Yoo PD (2017). Using the spanning tree of a criminal network for identifying its leaders. IEEE Transactions on Inf. Forensics Secur..

[7] Lü L, Zhang Y-C, Yeung CH, Zhou T (2011). Leaders in social networks, the delicious case. PLoS One.

[8] Leskovec J, Adamic LA, Huberman BA (2007). The dynamics of viral marketing. ACM Transactions on Web.

[9] Bovet, A., Morone, F. & Makse, H. A. Validation of twitter opinion trends with national polling aggregates: Hillary clinton vs donald trump. *Sci*. *Reports* **8**, 10.1038/s41598-018-26951-y (2018). 10.1038/s41598-018-26951-y PMC5989214 29875364

[10] Bovet, A. & Makse, H. A. Influence of fake news in twitter during the 2016 US presidential election. *Nat*. *Commun*. **10**, 10.1038/s41467-018-07761-2 (2019). 10.1038/s41467-018-07761-2 PMC6315042 30602729

[11] Zhao Tao, Huang Hong, Fu Xiaoming (2018). Identifying Topical Opinion Leaders in Social Community Question Answering. Database Systems for Advanced Applications.

[12] Temnikova, I. P., Castillo, C. & Vieweg, S. Emterms 1.0: A terminological resource for crisis tweets. In *ISCRAM* (2015).

[13] Imran, M., Mitra, P. & Castillo, C. Twitter as a lifeline: Human-annotated twitter corpora for nlp of crisis-related messages. *arXiv preprint arXiv:1605*.*05894* (2016).

[14] Imran, M., Elbassuoni, S., Castillo, C., Diaz, F. & Meier, P. Practical extraction of disaster-relevant information from social media. In *Proceedings of the 22nd International Conference on World Wide Web* - *WWW 13 Companion*, 10.1145/2487788.2488109 (ACM Press, 2013).

[15] Imran, M., Elbassuoni, S., Castillo, C., Diaz, F. & Meier, P. Extracting information nuggets from disaster-related messages in social media. In *Iscram* (2013).

[16] Helbing D (2015). Saving human lives: What complexity science and information systems can contribute. J. Stat. Phys..

[17] Perc, M., Ozer, M. & Hojnik, J. Social and juristic challenges of artificial intelligence. *Palgrave Commun*. **5**, 10.1057/s41599-019-0278-x (2019).

[18] Purohit, H., Castillo, C., Diaz, F., Sheth, A. & Meier, P. Emergency-relief coordination on social media: Automatically matching resource requests and offers. *First Monday* **19**, 10.5210/fm.v19i1.4848 (2013).

[19] Purohit H (2014). Identifying seekers and suppliers in social media communities to support crisis coordination. Comput. Support. Coop. Work. (CSCW).

[20] Basu, M. *et al*. Identifying post-disaster resource needs and availabilities from microblogs. In *Proceedings of the 2017 IEEE*/*ACM International Conference on Advances in Social Networks Analysis and Mining 2017* - *ASONAM 17*, 10.1145/3110025.3110036 (ACM Press, 2017).

[21] Gautam, B. & Basava, A. Automatic identification and ranking of emergency aids in social media macro community. *arXiv preprint arXiv:1810*.*11498* (2018).

[22] Costa PT, Mac Crae RR (1992). Neo personality inventory-revised (NEO PI-R).

[23] John, O. P. The “big five” factor taxonomy: Dimensions of personality in the natural language and in questionnaires. *Handb*. *personality: Theory research* (1990).

[24] Mohammad, S., Bravo-Marquez, F., Salameh, M. & Kiritchenko, S. SemEval-2018 task 1: Affect in tweets. In *Proceedings of The 12th International Workshop on Semantic Evaluation*, 1–17, 10.18653/v1/S18-1001 (Association for Computational Linguistics, New Orleans, Louisiana, 2018).

[25] Palguna, D. S., Joshi, V., Chakaravarthy, V. T., Kothari, R. & Subramaniam, L. V. Analysis of sampling algorithms for twitter. In *Proceedings of the Twenty*-*Fourth International Joint Conference on Artificial Intelligence*, *IJCAI 2015*, *Buenos Aires*, *Argentina*, *July 25*–*31*, *2015*, 967–973 (2015).

[26] Agarwal A, Toshniwal D (2019). Face off: Travel habits, road conditions and traffic city characteristics bared using twitter. IEEE Access.

[27] Agarwal A, Singh R, Toshniwal D (2018). Geospatial sentiment analysis using twitter data for uk-eu referendum. J. Inf. Optim. Sci..

[28] Agarwal, A., Gupta, B., Bhatt, G. & Mittal, A. Construction of a semi-automated model for FAQ retrieval via short message service. In *Proceedings of the 7th Forum for Information Retrieval Evaluation on* - *FIRE 15*, 10.1145/2838706.2838717 (ACM Press, 2015).

[29] 483 dead in kerala floods and landslides, losses more than annual plan outlay: Pinarayi vijayan, https://indianexpress.com/article/india/483-dead-in-kerala-floods-and-landslides-losses-more-than-annual-plan-outlay-pinarayi-vijayan-5332306/ (Accessed: 10-25-2018).

[30] Chief minister’s distress relief fund website, https://kerala.gov.in/cmsdistressrelieffund (Accessed: 10-25-2018).

[31] Ndrf saved 535 lives and evacuated more than 24,600 marooned people in flood-hit kerala, http://ndrf.gov.in/pressrelease/ndrf-saved-535-lives-and-evacuated-more-24600-marooned-people-flood-hit-kerala (Accessed: 10-25-2018).

[32] Center, n. h. national hurrican center tropical cyclone report, https://www.nhc.noaa.gov/data/#tcr (Accessed: 10-25-2018).

[33] Press, a. hurricane death toll in puerto rico more than doubles to 34, governor says, https://www.theguardian.com/world/2017/oct/03/puerto-rico-new-death-toll-hurricane-maria-trump-visit (Accessed: 10-25-2018).

[34] Team v. west sumatra provincial government sends 1 ton rendang for lombok earthquake victims, https://www.viva.co.id/berita/nasional/1062665-pemprov-sumbar-kirim-satu-ton-rendang-untuk-korban-gempa-lombok (Accessed: 10-25-2018).

[35] Dani, s. aceh government collects funds for lombok earthquake victims, rp. 300 million already collected, https://aceh.tribunnews.com/2018/08/07/pemerintah-aceh-galang-dana-untuk-korban-gempa-lombok-sudah-terkumpul-rp-300-juta (Accessed: 10-25-2018).

[36] Rms estimates insured losses from typhoon jebi could reach $5.5 billion, https://www.insurancejournal.com/news/international/2018/09/14/501390.htm (Accessed: 10-25-2018).

[37] Pei, S., *et al*. Searching for superspreaders of information in real-world social media. *Sci*. *Reports* **4**, 5547, 10.1038/srep05547, 1405.1790 (2014). 10.1038/srep05547 PMC4080224 24989148

[38] Bastian, M., Heymann, S. & Jacomy, M. Gephi: an open source software for exploring and manipulating networks. In *Third international AAAI conference on weblogs and social media* (2009).

[39] Blanks, B. With lessons learned from maria, direct relief, facebook refine crisis response tools in puerto rico. *ReliefWeb* (2018).

[40] Defence minister of india :kerala flood relief efforts, https://tinyurl.com/yxnfzqmy (Accessed: 01-10-2019).

[41] News, U. & Report, W. Best states rankings: Measuring outcomes for citizens using more than 70 metrics. *U*.*S*. *News World Rep*. (2019).

[42] Foundation, S. S. E. Smart cities index: A tool for evaluating cities. *Shakti Sustain*. *Energy Foundation* (2018).

[43] Song, S. & Meng, Y. Classifying and ranking microblogging hashtags with news categories. In *2015 IEEE 9th International Conference on Research Challenges in Information Science* (*RCIS*), 540–541, 10.1109/RCIS.2015.7128928 (2015).

[44] Saqaf, S. M. Flood alert issued in salem, namakkal districts. *The Hindu* (2018).

[45] McNoldy, B. & Samenow, J. Tropical storm warning for n.c. outer banks as irma may soon form. *The Wash*. *Post* (2017).

[46] Center, N. S. F. Nasa’s gpm satellite sees jebi as another tropical threat to japan. *Am*. *Assoc*. *for Adv*. *Sci*. (2018).

[47] Group, S. B. Tropical storm maria could follow hurricane irma’s path. *ABC7 WJLA* (2017).

[48] Al-Garadi MA (2018). Analysis of online social network connections for identification of influential users: Survey and open research issues. ACM Comput. Surv..

[49] Kempe, D., Kleinberg, J. & Tardos, É. Maximizing the spread of influence through a social network. In *Proceedings of the ninth ACM SIGKDD international conference on Knowledge discovery and data mining* - *KDD 03*, 10.1145/956750.956769 (ACM Press, 2003).

[50] Judge TA, Bono JE, Ilies R, Gerhardt MW (2002). Personality and leadership: A qualitative and quantitative review. J. Appl. Psychol..

[51] Li, S., Li, W., Cook, C., Zhu, C. & Gao, Y. Independently recurrent neural network (indrnn): Building A longer and deeper RNN. *CoRR* abs/1803.04831, 1803.04831 (2018).

[52] Zhou, P. *et al*. Attention-based bidirectional long short-term memory networks for relation classification. In *Proceedings of the 54th Annual Meeting of the Association for Computational Linguistics* (*Volume 2: Short Papers*), 207–212, 10.18653/v1/P16-2034 (Association for Computational Linguistics, 2016).

[53] Miyato, T., Dai, A. M. & Goodfellow, I. Adversarial Training Methods for Semi-Supervised Text Classification. *ArXiv e*-*prints* 1605.07725.

[54] Yang, Z. *et al*. Hierarchical attention networks for document classification. In *Proceedings of the 2016 Conference of the North American Chapter of the Association for Computational Linguistics: Human Language Technologies*, 1480–1489, 10.18653/v1/N16-1174 (Association for Computational Linguistics, 2016).

[55] Zubiaga A (2017). Towards real-time, country-level location classification of worldwide tweets. IEEE Transactions on Knowl. Data Eng..

[56] Zhang Y, Maciejewski R (2017). Quantifying the visual impact of classification boundaries in choropleth maps. IEEE Transactions on Vis. Comput. Graph..

[57] Countries, http://www.naturalearthdata.com/http//www.naturalearthdata.com/download/10m/cultural/ne_10m_admin_0_countries.zip (Accessed: 10-25-2018).

[58] States, regions and municipalities, http://www.naturalearthdata.com/http//www.naturalearthdata.com/download/10m/cultural/ne_10m_admin_1_states_provinces.zip (Accessed: 10-25-2018).

[59] Us counties, http://www2.census.gov/geo/tiger/GENZ2015/shp/cb_2015_us_county_500k.zip (Accessed: 10-25-2018).

[60] Us counties, http://www2.census.gov/geo/tiger/GENZ2015/shp/cb_2015_us_state_5m.zip (Accessed: 10-25-2018).

[61] Knaub AV, Henderson C, Fisher KQ (2018). Finding the leaders: an examination of social network analysis and leadership identification in stem education change. Int. J. STEM Educ..

[62] Badenes, H. *et al*. System u: Automatically deriving personality traits from social media for people recommendation. In *Proceedings of the 8th ACM Conference on Recommender Systems*, *RecSys* ’*14*, 373–374, 10.1145/2645710.2645719 (ACM, New York, NY, USA, 2014).

[63] Pennington, J., Socher, R. & Manning, C. D. Glove: Global vectors for word representation. In *Empirical Methods in Natural Language Processing* (*EMNLP*), 1532–1543 (2014).

[64] Warriner AB, Kuperman V, Brysbaert M (2013). Norms of valence, arousal, and dominance for 13,915 english lemmas. Behav. Res. Methods.

[65] Bradley, M. M., Lang, P. J., Bradley, M. M. & Lang, P. J. Affective norms for english words (anew): Instruction manual and affective ratings (1999).

[66] Holme, P. Rare and everywhere: Perspectives on scale-free networks. *Nat*. *Commun*. **10**, 10.1038/s41467-019-09038-8 (2019). 10.1038/s41467-019-09038-8 PMC6399274 30833568

[67] Demšar J (2013). Orange: Data mining toolbox in python. J. Mach. Learn. Res..

