# Supplementary material for: Identifying Leadership Characteristics from Social Media Data during Natural Hazards using Personality Traits
Source: Sci Rep. 2020 Feb 14;10:2624. doi: 10.1038/s41598-020-59086-0 (PMC7021906; doi:10.1038/s41598-020-59086-0)
Supplement: Supplementary file 1 — Supplementary Information. [file 41598_2020_59086_MOESM1_ESM.pdf]

Natural Hazard	Geographical Regions	Frequency	Frequency Distributions			
			Resource	Casualty	News	Sympathy
Kerala Floods	Madhya Pradesh	360,313	098,255	108,923	057,379	095,744
	Delhi	162,531	044,293	049,134	025,910	043,183
	Maharashtra	152,655	041,652	046,294	024,196	040,506
	Tamil Nadu	149,497	040,515	045,329	023,719	039,931
	Karnataka	129,470	035,329	039,138	020,582	034,415
Hurricane Florence	Rooks, Kansas	074,369	018,028	028,891	016,469	010,975
	New York	018,367	004,419	007,148	004,025	002,774
	Tulare, California	017,528	004,388	006,853	003,700	002,584
	Washington, DC	016,125	003,907	006,373	003,454	002,391
	Los Angeles, California	013,351	003,214	005,247	002,922	001,967
Hurricane Irma	Rooks, Kansas	013,074	015,020	028,380	036,767	022,903
	Miami-Dade, Florida	055,184	008,033	015,390	019,434	012,323
	New York	037,311	005,441	010,319	013,192	008,358
	Tulare, California	027,325	003,932	007,620	009,621	006,152
	Fulton, Georgia	026,366	003,856	007,320	009,374	005,816
Hurricane Maria	Rooks, Kansas	154.870	023,671	045.670	060,779	024,747
	New York	071,489	010,871	021,138	028,074	011,405
	Tulare, California	048,915	007,266	014,376	019,348	007,926
	Los Angeles, California	043,885	006,673	012,918	017,244	007,050
	Washington, DC	038,840	005,874	011,392	015,381	006,191
Iran–Iraq Earthquake	Rooks, Kansas	170,811	059,749	084,117	018,619	008,310
	Westminster	088,375	030,950	043,733	009,507	004,173
	Yazd	041,692	014,745	020,458	004,499	001,990
	Lamb, Texas	038,890	013,452	019,324	004,206	001,905
	Washington, DC	038,768	013,792	018,933	004,180	001,861
Lombok Earthquake	Kalimantan Tengah	041,620	015,402	014,713	007,564	003,936
	Sarawak	030,609	011,245	010,834	005,586	002,928
	North West	024,729	009,215	008,784	004,365	002,358
	Jakarta Raya	015,151	005,584	005,422	002,645	001,499
	Rooks, Kansas	014,707	005,418	005,380	002,555	001,354
Typhoon Mangkhut	Cavite	010,630	001,996	004,624	003,211	000,792
	Kwai Tsing	008,479	001,499	003,792	002,602	000,586
	Rooks, Kansas	008,350	001,496	003,688	002,566	000,598
	Westminster	005,349	000,996	002,300	001,654	000,398
	Manila	004,183	000,760	001,795	001,326	000,299
Typhoon Jebi	Rooks, Kansas	136,143	057,021	045,865	019,531	013,725
	New York	049,610	020,753	016,924	006,826	005,107
	Westminster	040,195	016,943	013,513	005,596	004,143
	Tulare, California	038,153	015,785	013,205	005,391	003,772
	Los Angeles, California	033,266	013,805	011,346	004,705	003,410

Table:1 Top k Geographical Polygon Regions

Year	Hazards	Labels Distribution				References
		Resource	Casuality	News	Sympathy	
2011 - 2014	Tornado	2463	275	0	1668	13, 15, 41
2011 - 2014	Typhoon	507	556	0	7381	13, 15, 41
2012 - 2017	Hurricane	1264	1268	0	735	13, 14, 41–43
2013 - 2017	Earthquake	1962	2719	0	1966	13, 41, 42, 44, 45
2013 - 2017	Floods	577	1643	0	257	13, 42, 45
2014	Volcano	161	0	0	33	13
2014	Missing	10	0	0	59	13
2014	Biological	698	960	856	52	13
2015	Cyclone	389	464	3	339	13
2014 - 2015	Landslides	0	0	4918	0	13
**2011 - 2017**	**Total**	**8031 (23.49 %)**	**7885 (23.06 %)**	**5774 (16.89 %)**	**12490 (36.54 %)**	

Table 2: Detailed overview of the data sources used in this study.

References:

[13] Imran, M., Mitra, P. & Castillo, C. Twitter as a lifeline: Human-annotated twitter corpora for nlp of crisis-related messages. arXiv preprint arXiv:1605.05894 (2016).

[14] Imran, M., Elbassuoni, S., Castillo, C., Diaz, F. & Meier, P. Practical extraction of disaster-relevant information from social media. In Proceedings of the 22nd International Conference on World Wide Web - WWW 13 Companion, DOI: 10.1145/2487788.2488109 (ACM Press, 2013).

[15] Imran, M., Elbassuoni, S., Castillo, C., Diaz, F. & Meier, P. Extracting information nuggets from disaster-related messages in social media. In Iscram (2013).

[41] Nguyen, D. T., Ofli, F., Imran, M. & Mitra, P. Damage assessment from social media imagery data during disasters. In Proceedings of the 2017 IEEE/ACM International Conference on Advances in Social Networks Analysis and Mining 2017, 569–576 (ACM, 2017).

[42]. Alam, F., Ofli, F. & Imran, M. Crisismmd: Multimodal twitter datasets from natural disasters. In Twelfth International AAAI Conference on Web and Social Media (2018).

[43]. Alam, F., Ofli, F., Imran, M. & Aupetit, M. A twitter tale of three hurricanes: Harvey, irma, and maria. arXiv preprint arXiv:1805.05144 (2018).

[44]. Alam, F., Joty, S. & Imran, M. Domain adaptation with adversarial training and graph embeddings. arXiv preprint arXiv:1805.05151 (2018).

[45]. Alam, F., Joty, S. & Imran, M. Graph based semi-supervised learning with convolution neural networks to classify crisis related tweets. In Twelfth International AAAI Conference on Web and Social Media (2018).
